# Retrospective Analysis of Clinical and Cost Outcomes Associated with Methicillin-Resistant *Staphylococcus aureus* Complicated Skin and Skin Structure Infections Treated with Daptomycin, Vancomycin, or Linezolid

**DOI:** 10.4061/2011/347969

**Published:** 2010-12-29

**Authors:** Bradley M. Wright, Edward H. Eiland

**Affiliations:** ^1^PharmD, BCPS Department of Pharmacy Practice, Auburn University Harrison School of Pharmacy, 650 Clinic Drive, Room 2100, Mobile, AL 36688, USA; ^2^PharmD, MBA, BCPS AQ-ID, Huntsville Hospital Department of Pharmacy, 101 Sivley Road, Huntsville, AL 35801, USA

## Abstract

*Objective*. The objective of this analysis was to compare clinical and cost outcomes associated with patients who had suspected or documented methicillin-resistant *Staphylococcus aureus* (MRSA) infections treated with daptomycin, vancomycin, or linezolid in complicated skin and skin structure infections (cSSSIs). *Design*. This was a retrospective analysis conducted from February to June of 2007. Appropriate data was collected, collated, and subsequently evaluated with the purpose of quantifying length of stay, antibiotic therapy duration, clinical cure rates, adverse drug events, and cost of hospitalization. *Results*. All 82 patients included in the analysis experienced clinical cure. The duration of antibiotic therapy was similar among the three groups yet the length of hospitalization was slightly shorter in the daptomycin group. *Conclusions*. The incidence of resistant staphylococcal infections is increasing; therefore, judicious use of MRSA active agents is paramount. Future studies are necessary to determine if MRSA treatment options can be stratified based on the severity of the infectious process.

## 1. Introduction

Skin and skin structure infections (SSSIs) are common within the hospital setting as well as in the community. Some common examples of SSSIs include abscesses, cellulitis, diabetic foot infections, and surgical site infections. Oral antibiotics can often be used to safely manage many SSSIs; however, complicated SSSIs (cSSSIs) may be life threatening and often involve deeper layers of skin and more severe symptoms which will often necessitate hospitalization, intravenous antibiotics, and may also require surgical intervention. 

 Although a variety of gram-positive and gram-negative pathogens may cause SSSIs, methicillin-resistant *Staphylococcus aureus* (MRSA) has emerged as the most common pathogen [1, 2] A study of 265 community hospitals in the Southeastern United States found the prevalence of MRSA as the causative pathogen for surgical site infections increased from 0.12 infections per 100 procedures to 0.23 infections per 100 procedures from 2000 to 2005 [3] In addition, a retrospective analysis of 288 patients managed with operative debridement for SSSIs found that the incidence of MRSA increased from 34 percent to 77 percent from 2000 to 2006. The percentage of MRSA isolates with an MIC ≤ 0.5 mcg/mL to vancomycin decreased from 100 percent in 2000 to 62 percent in 2006 [4]. This increasing prevalence of MRSA infection within the hospital and the community further complicates the treatment of SSSIs as MRSA infections are also known to be associated with an increased incidence of bacteremia, septic shock, amputation and overall mortality [5]. 

 This increasing trend of resistance has not only been noted within the realm of SSSIs but similar trends have been observed for MRSA in general. Data collected by the National Nosocomial Infections Surveillance System showed that the percentage of MRSA isolates in intensive care units increased from 36 percent in 1992 to 64 percent in 2003 [6]. The economic impact associated with the increasing number of total MRSA infections is vast, and these resistant infections are well established as prolonging hospital length of stay and increasing total healthcare costs [5, 7, 8]. This impact is not a new phenomena considering that epidemiologic data as far back as the 1980s show that the mean length of stay (LOS) associated with MRSA infection is about twice as long as that for methicillin-susceptible *S. aureus* (MSSA) infection [5] The cost of hospitalization for a patient with infections due to MRSA is 2.5 times more than for patients with infections caused by MSSA [7]. Recent data has also provided similar observations as Engemann noted a higher 90-day mortality rate (odds ratio 3.4) compared with MSSA in patients with a surgical site infection caused by MRSA [8]. 

 The economic impact of hospitalization is a major concern; however, this is not the only burden when discussing MRSA infection. Reports of decreased susceptibility of *S. aureus*, including MRSA, to vancomycin (Vancocin—Eli Lilly), and also the associated mortality and cost consequences of resistance, highlight the need for alternative antibiotics [9, 10]. One such alternative agent, daptomycin (Cubicin—Cubist), was approved by the US Food and Drug Administration (FDA) in September 2003 for the treatment of cSSSIs infections due to susceptible strains of S. aureus (including MRSA), *S. pyogenes*, *S. agalactiae*, *S. dysgalactiae* subspecies Equisimilis and *Enterococcus faecalis* (vancomycin-susceptible strains only). Another alternative agent, linezolid (Zyvox - Pfizer), was approved by the FDA in 2000 for the treatment of patients with infections caused by vancomycin-resistant *Enterococcus faecium* (VREF) or methicillin-resistant *Staphylococcus aureus* (MRSA).

## 2. Objective

The objective of this analysis was to compare clinical and cost outcomes of patients who had suspected or documented MRSA cSSSIs treated with daptomycin, vancomycin, or linezolid. Moreover, this study compared hospital lengths of stay between these three therapeutic options.

## 3. Methods

This retrospective analysis was conducted from February to June 2007 and took place at Huntsville Hospital, an 881-bed level 1 trauma and regional referral center located in Northeast Alabama. No external funding was sought for this analysis. Review of medical records of hospitalized patients with cSSSIs that were treated with vancomycin, linezolid, or daptomycin was performed after approval by the Institutional Review Board. Eligible patients included adults aged 17 years or greater with risk factors for MRSA infections who were admitted to the hospital with a clinical diagnosis of cSSSIs defined as cellulitis, skin abscess, decubitus ulcer, infected amputation stump, or surgical site infection. Patients were excluded from the analysis if they exhibited any of the following characteristics: pneumonia, bacteremia, endocarditis, osteomyelitis, patients with MRSA MIC's to vancomycin of ≥2 mcg/mL (at the time of study completion this institution had no MICs ≥2) or documented resistance to daptomycin or linezolid, patients with prosthetic valves, patients with a vancomycin, daptomycin, or linezolid allergy, or pregnant, lactating, neutropenic, or HIV-infected patients. Antibiotic therapy was left to the discretion of the prescribing physician; however, this choice could have been influenced by the presence of a criterion for linezolid use at this institution. Incision and drainage and/or debridement were not performed on these patients as this is consistent with previous cSSSIs trials previously completed [11, 12]. 

 Data collected included gender, age, weight, antibiotic allergies, dialysis status, hospital length of stay, additional therapies, and microbiological culture and susceptibility results. Antibiotic therapy data collected included antibiotic, dose, route, frequency, indication, and total number of doses. Pertinent lab data collected included complete blood cell count and basic metabolic panel or complete metabolic panel. 

 The primary study endpoints were: duration of antibiotic therapy, total hospital length of stay, total stay in the intensive care unit, total cost of hospitalization for each patient, and antibiotic-associated adverse reactions and adverse drug events. 

 None of the study endpoints were stratified by pathogen as the only pathogen providing a positive culture result in these patients was MRSA seen in 10/26 in the daptomycin group, 8/28 in the vancomycin group, and 8/28 in the linezolid group. Patients were captured based on CPT code for cellulitis, abscess, decubitus ulcer, infected amputation stump or surgical site infection and were treated based on suspected or documented MRSA infection. While only 26/82 patients actually had a positive culture for MRSA, each patient had risk factors for MRSA infection and no other organisms were identified from these cultures. 

 The average dose for daptomycin was 576 mg per day; linezolid was 1200 mg per day; and vancomycin was 2430 mg per day in order to achieve targeted vancomycin troughs of 15–20 mg/dL. The vancomycin, daptomycin, and linezolid therapies were discontinued based on clinical cure/improvement represented by the patient being afebrile with a WBC ≤ 10,000/mm^3^, no bandemia, and a visibly resolved cSSSIs noted by the physician. Patient's must also have meet criteria for hospital discharge and completed their course of IV antibiotics while hospitalized. Patients could be discharged with or without oral antimicrobial therapy. Repeat blood cultures at the end of therapy were not collected to determine microbiological cure as all patients met the endpoint described as clinical cure. Adverse drug events were determined by physicians, nurses, and pharmacists. The Naranjo score was calculated for each probable ADR [13, 14].

## 4. Results

A total of 82 patients were included in this analysis with 26 receiving daptomycin, 28 vancomycin, and 28 linezolid. Fifty-seven percent of patients were male and the mean age ranged from 54 years (vancomycin) to 60 years (linezolid). Thirty-two percent (26/82) of the evaluated patients had a culture positive for MRSA and a slight majority, 38 percent (10/26) were in the daptomycin group. Cellulitis was the most common infection in each group and accounted for 67 percent of all infections. All patients included in this analysis were discharged from the hospital and achieved clinical cure/improvement per the guidelines above. 

**Figure 1:**
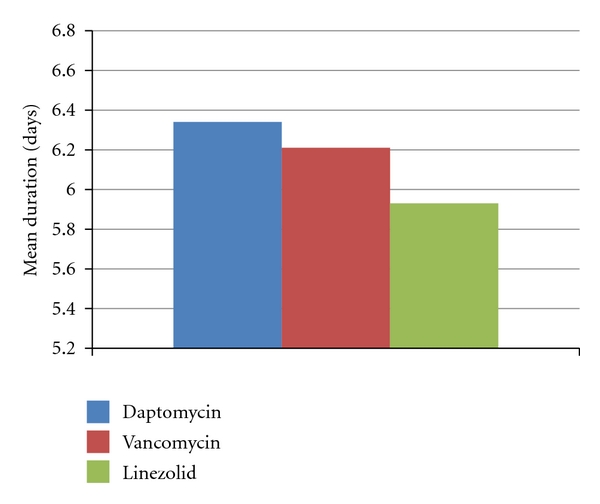
Mean duration of antibiotic therapy.

Figure 1 displays the duration of antibiotic therapy results. The mean duration of antibiotic therapy was similar among the three groups, ranging from 5.93 days for linezolid to 6.21 days for vancomycin to 6.34 days for daptomycin. However, the mean number of antibiotic doses varied significantly among the three groups. The daptomycin group had the fewest mean number of doses with 5.5 doses, followed by vancomycin with 8.1 doses, and linezolid with 11.1 doses. Daptomycin is dosed once daily or if CrCl < 30 mL/min every 48 hours, vancomycin dosing is dependent upon and varies based on renal function, and linezolid dosing is standardized to every 12 hours regardless renal function. Based on these dosing regimens, the result regarding mean number of doses would be expected. Additionally, of the 28 patients in the linezolid group, there were a total of 128 intravenous (IV) therapy days and 38 oral (PO) therapy days. A total of five patients were switched from IV to PO during the course of linezolid therapy. 

**Figure 2:**
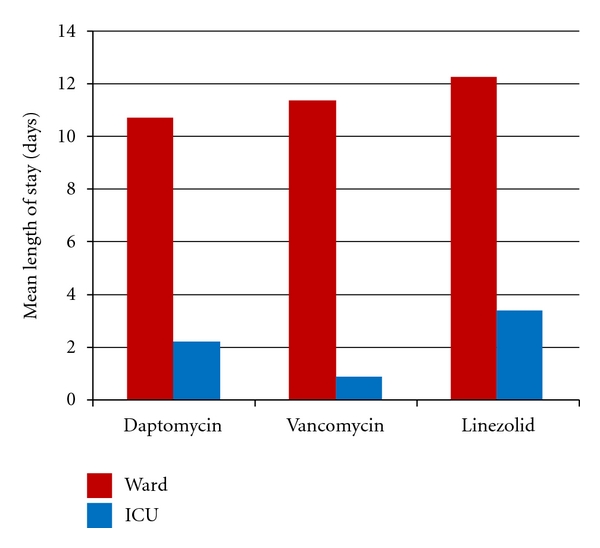
Mean length of hospitalization.

Figure 2 portrays the length of hospitalization results. Mean length of hospitalization was shortest in the vancomycin group at 12.3 days compared with 12.9 days and 15.7 days for the daptomycin and linezolid groups, respectively. Length of stay on the ward and ICU was also compared between the agents. Length of stay on the ward was shortest for the daptomycin group at 10.6 days followed by the vancomycin group at 11.2 days and then the linezolid group at 12.4 days. Length of stay in the ICU was shortest for the vancomycin group at 0.9 days followed by daptomycin at 2 days and lastly linezolid at 3.4 days. 

**Figure 3:**
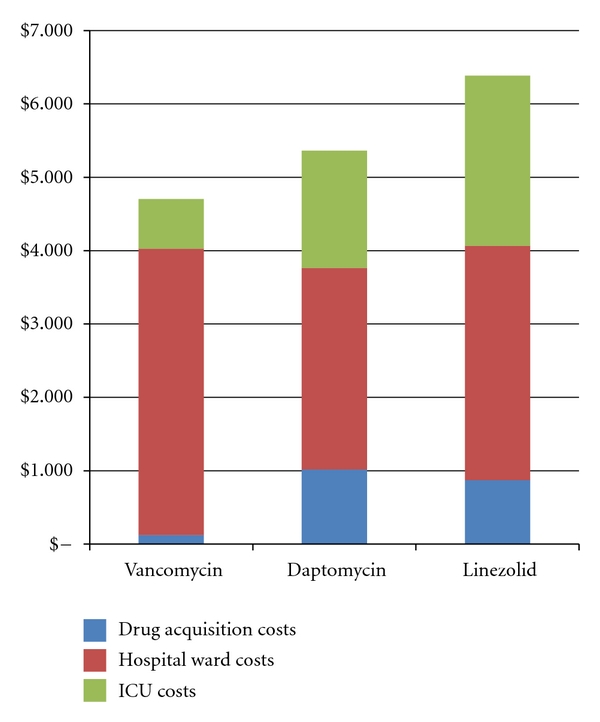
Cost of therapy.

 Cost of therapy is portrayed in Figure 3. The mean total cost of therapy per patient was $4703.57 for vancomycin, $5364.48 for daptomycin, and $6384.79 for linezolid. Drug acquisition and administration costs, including drug levels for vancomycin, were $123.78 for vancomycin, $1017.17 for daptomycin, and $872.29 for linezolid. Hospital ward costs for vancomycin, daptomycin, and linezolid were $3904.04, $2749.19, and $3192.57, respectively, while ICU costs were $675.75, $1598.12, and $2319.93, respectively. 

 No patients treated with daptomycin or linezolid experienced an antibiotic-associated adverse drug event (ADE). One patient in the vancomycin group experienced Red-man's syndrome after the first dose of vancomycin requiring an additional medication (diphenhydramine 25 mg IVP X 1 dose) to treat this adverse drug reaction (ADR). The cost of this additional medication was nominal at $0.25 yet was still added into the cost of the vancomycin therapy. The Naranjo score was calculated as a 7 designating the event as a probable adverse drug reaction [13, 14]. The patient was continued on the vancomycin therapy yet the infusion rate was slowed to not exceed 1 gram per hour per the recommendations in the package insert.

## 5. Discussion

The principle causative pathogen in cSSSIs, MRSA, has become well known. This multi-drug resistant organism is associated with a significant impact on morbidity, mortality, length of hospitalization, and cost of care. In the present study, MRSA was responsible for 26/82 (32 percent) of cSSSIs, however, each patient had risk factors for MRSA infection and no other organisms were identified. This is lower than the 77 percent in 2006 reported by Awad et al.; however, this may reflect a generally sicker population in that study since patients required surgical debridement for their cSSSIs [4]. Further complicating this picture is the recent emergence of MRSA with decreased susceptibility to vancomycin, further justifying the potential demand for alternative antimicrobial agents [15]. Two such alternative agents are linezolid and daptomycin which both offer specific therapeutic advantages over vancomycin in certain infectious processes. 

 A primary objective of this study was to assess patient outcomes and the associated costs related to drug acquisition, administration, and per diem hospital costs for daptomycin compared with vancomycin and linezolid. The mean total cost for daptomycin therapy was intermediate between vancomycin and linezolid due to the decreased cost for the patient hospital stay on the ward associated with daptomycin compared with vancomycin and linezolid which countered the increased acquisition cost of the drug. All agents evaluated were also associated with a similar and a relatively short duration of antibiotic therapy in the present study. The duration noted in this analysis was shorter than that recommended in the package inserts of each respective agent for cSSSIs. Length of hospital stay was also compared and found to be similar in the daptomycin and vancomycin groups, while longer in the linezolid group. This finding is interesting considering the oral availability of linezolid and the ability to transition to the oral equivalent with this agent. Shorter length of intensive care unit stay in the vancomycin group contributed to the overall lower cost of drug therapy compared with daptomycin and linezolid. In the present study, clinical cure/improvement documented post treatment was represented by the patient being afebrile with a WBC < 10,000 with no bandemia and a visibly resolved cSSSIs. All patients in this study met the definition for clinical cure/improvement. 

 Other studies to investigate these therapeutic alternatives in cSSSIs were found using an English-language Medline search. Weigelt [16] and colleagues describe a study in which 1180 patients with a cSSSIs were treated with linezolid 600 mg IVPB and PO or vancomycin 1 gram IVPB every 12 hours. Duration of treatment with linezolid was 11.8 days and was longer than that of vancomycin at 10.9 days; *P* < .004. Duration of IV treatment was also shorter for linezolid (4 versus 9 days; *P* < .0001). No significant difference was found between the clinical cure rates between linezolid (92.2 percent) versus vancomycin (88.5 percent). Modified intent to treat microbiologic cure of MRSA of linezolid was 71 percent and vancomycin was 55.1 percent (*P* = .002). MSSA microbiological cure was found to be 73 percent versus 66.4 percent (*P* = .264) [16]. 

 Itani describes the health outcomes resulting from this large study as well as duration of IV treatment and weekly discharges [17]. With regards to the ITT population, the mean initial LOS was significantly shorter in the linezolid arm (6.7 days versus 9.4 days, *P* < .01). Following initial hospitalization, more patients receiving linezolid were discharged from the hospital than those receiving vancomycin (*P* < .0001). The rates of infection-related readmission between the two groups was comparable for all study populations (*P* > .05) and finally, fewer IV antibioitic treatment days were needed in patients receiving linezolid compared with those receiving vancomycin (*P* < .0001) [17]. 

 Another evaluation of this data among United States subjects also found clinical and cost advantages for patients treated with linezolid [18]. Mean cost for ITT population patients treated with linezolid versus vancomycin was $4865 versus $5738, respectively, (*P* = .017), and in the MRSA population specifically the cost was $4481 versus $6006 for linezolid and vancomycin, respectively (*P* = .041). Total costs in this study included all costs incurred by the patients during hospitalization and following discharge [18]. 

 Stevens and colleagues [19] published a study of 460 patients with presumed SSSIs MRSA infection that were randomized to linezolid 600 mg IVPB/PO or vancomycin 1 gram IVPB every 12 hours for 7 days or longer. Clinical cure (intent to treat) was 64.6 percent in the linezolid group versus 62.1 percent in the vancomycin group and 69.8 percent and 74.4 percent, respectively, (*P* value not provided) when looking only at patients with MRSA [19]. 

 Previous trials have also shown daptomycin to be clinically efficacious and exhibits a trend toward positive economic outcomes when compared to vancomycin. Specifically, daptomycin was shown to provide a more rapid clinical response than vancomycin in an open-label study of 265 patients with cSSSIs [11]. Additionally, hospital costs were noted to be significantly lower in the daptomycin group in this study as patients in the daptomycin group were found on average to need 3 days less of antimicrobial therapy and a significantly higher proportion of patients achieved complete resolution of their infections. Other clinical trial data show a trend toward decreased time to resolution of symptoms with 63% of daptomycin-treated patients and 33% of the comparator group requiring only 4–7 days of therapy [11]. In addition to these findings, In vitro and animal data seem to indicate that daptomycin is rapidly bactericidal when compared with vancomycin [11]. 

 The efficacy and safety of daptomycin in cSSSIs was demonstrated in 2 clinical trials involving a total of 1092 patients. Daptomycin 4 mg/kg IVPB every 24 hours for 7–14 days was compared with penicillinase-resistant penicillins 4–12 grams IVPB per day or vancomycin 1 gram IVPB every 12 hours and clinical success was achieved in 83.4 percent and 84.2 percent of the daptomycin and comparator patients. Clinical success was achieved in 85.9 percent and 87 percent, respectively, of those infected with MSSA at baseline, while clinical success was achieved in 75 percent and 69.4 percent, respectively, of those infected with MRSA at baseline. The frequency and distribution of adverse events were similar among both treatment groups in these trials [12]. 

 Limitations of the present analysis include the retrospective method in which it was performed, prohibiting a definitive conclusion regarding causality. Additionally, the clinical effectiveness of vancomycin was less defined during the study period with regard to the developing data describing an MRSA MIC creep to this agent. Furthermore, at the time in which the study was conducted, the institutions Pharmacy and Therapeutics Committee positioned vancomycin as the first-line agent for cSSSIs ahead of daptomycin and linezolid as to allow vancomycin to serve as the most common empiric therapy resulting in patients having received vancomycin prior to receiving alternate agents such as daptomycin and linezolid.

## 6. Conclusion

The incidence of resistant staphylococcal infections is increasing; therefore, the judicious use of antibiotics, such as daptomycin, linezolid, and vancomycin, with good activity against MRSA, is paramount. This study showed similar clinical and cost outcomes with the use of daptomycin when compared with vancomycin and linezolid in cSSSIs for a similar patient population. In addition, while daptomycin showed a slightly greater overall duration of therapy it was well tolerated and showed an overall decreased length of stay in the ward comparatively which proved to be financially advantageous considering that per diem hospital costs accounts were the most expensive healthcare resource in this study population. The retrospective design of this analysis further justifies the conclusion that future studies are necessary to determine if MRSA treatment options can be stratified based on the severity of the infectious process as to ensure that the duration of a patient's hospitalization is optimized.

## Figures and Tables

**Table 1:** Demographics.

	Daptomycin	Vancomycin	Linezolid
	(*n* = 26)	(*n* = 28)	(*n* = 28)
Gender			
Female	14	7	14
Male	12	21	14
Age (years)			
Mean	59	54	60
Range	18–88	17–86	19–93
Positive culture for MRSA	10	8	8
Infection type			
Cellulitis	16	21	18
Decubitus ulcer	6	3	0
Abscess	2	4	9
Surgical site	2	0	1

Values are presented as *n*. MRSA: Methicillin-resistant *Staphylococcus aureus.*
